# Wet‐Spun PEDOT/CNT Composite Hollow Fibers as Flexible Electrodes for H_2_O_2_ Production**

**DOI:** 10.1002/celc.202100237

**Published:** 2021-05-04

**Authors:** Qing Cui, Daniel Josef Bell, Siqi Wang, Mojtaba Mohseni, Daniel Felder, Jonas Lölsberg, Matthias Wessling

**Affiliations:** ^1^ RWTH Aachen University Chemical Process Engineering Forckenbeckstr. 51 52074 Aachen Germany; ^2^ DWI Leibniz-Institute for Interactive Materials Forckenbeckstr. 50 52074 Aachen Germany

**Keywords:** carbon nanotubes, electrochemistry, PEDOT, polymers, oxygen reduction reaction

## Abstract

The electrochemical synthesis of hydrogen peroxide (H_2_O_2_) using the oxygen reduction reaction (ORR) requires highly catalytic active, selective, and stable electrode materials to realize a green and efficient process. The present publication shows for the first time the application of a facile one‐step bottom‐up wet‐spinning approach for the continuous fabrication of stable and flexible tubular poly(3,4‐ethylene dioxythiophene) (PEDOT : PSS) and PEDOT : PSS/carbon nanotube (CNT) hollow fibers. Additionally, electrochemical experiments reveal the catalytic activity of acid‐treated PEDOT : PSS and its composites in the ORR forming hydrogen peroxide for the first time. Under optimized conditions, the composite electrodes with 40 wt % CNT loading could achieve a high production rate of 0.01 mg/min/cm^2^ and a current efficiency of up to 54 %. In addition to the high production rate, the composite hollow fiber has proven its long‐term stability with 95 % current retention after 20 h of hydrogen peroxide production.

## Graphical abstract

**A one‐step fabrication platform** for the continuous production of a hollow acid‐treated poly(3,4‐ethylene dioxythiophene)/carbon nanotube (PEDOT/CNT) composite is developed based on a 3D‐printed core‐shell spinneret for bottom‐up spinning. The composite electrode shows an excellent performance in the oxygen reduction reaction, with a high activity and selectivity towards hydrogen peroxide formation.

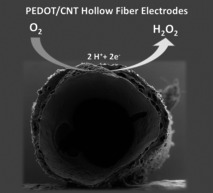

## 1 Introduction

The electrochemical conversion of molecular oxygen in the oxygen reduction reaction (ORR) poses a green alternative towards the sustainable production of hydrogen peroxide.[^1^, ^2^] Compared to the traditional anthraquinone process, the ORR is a one‐step production process with fewer waste products, lower energy demand, and production costs.^[3]^

Dissolved oxygen can be reduced in acidic aqueous solution on the cathode using two electrons (two‐electron pathway) forming hydrogen peroxide (1). Two side reactions, namely the further reduction of hydrogen peroxide to water (2) or the direct reduction to water in the four‐electron pathway (3), can hinder the efficient electrochemical synthesis of hydrogen peroxide.

(1) $$O_{2}+2H^{+}+2e^{-}\to H_{2}O_{2}$$

(2) $${H_{2}O}_{2}+2H^{+}+2e^{-}\;\to 2\;H_{2}O$$

(3) $$O_{2}+4H^{+}+4e^{-}\to {2\;H}_{2}O$$

To favor the two‐electron transfer reaction and increase the selectivity and production rate of hydrogen peroxide, the development of electrode materials with high catalytic activity and selectivity is paramount. Due to the high costs of noble metal catalysts for the ORR, massive efforts have been made to develop affordable metal‐free catalysts with high performance, good long‐term stability, and robust mechanical properties, enabling their application beyond lab‐scale experiments.[^1^, ^2^, ^4^, ^5^]

Besides the carbon‐based materials like carbon nanotubes (CNTs) and graphene, which are extensively investigated and known to be suitable catalysts for the two electron‐pathway, conducting polymers are a new catalyst class with great potential for the ORR.[^6^, ^7^] Amongst the conducting polymers, poly (3,4‐ethylene dioxythiophene) (PEDOT) is the most investigated for ORR.[^8^, ^9^, ^10^, ^11^, ^12^] The inherent advantage of this material contributes to its high chemical stability, high mechanical flexibility, and its unique‐combined electric and ionic conductivity.^[13]^ Furthermore, PEDOT : PSS swells in aqueous media, forming a permeable 3D network enabling the coincidence of electrons, ions and reacting molecules.^[14]^

Winther‐Jensen *et al*. reported the catalytic ability of PEDOT towards the four‐electron transfer in the ORR, showing a catalytic activity comparable to Platinum.^[15]^ A second study by Winther‐Jensen *et al*. indicates that the catalytic activity of PEDOT electrodes and their ability to perform either the two‐ or four‐electron pathway are strongly affected by the preparation method.^[16]^ Since then, different experimental and theoretical studies highlight the ability of PEDOT‐based electrodes to reduce oxygen. However, the majority of research is focused on the four‐electron pathway.[^2^, ^9^, ^12^, ^17^, ^18^, ^19^] In the first systematic study on the hydrogen peroxide formation using PEDOT in 2019, Mitraka *et al*. analyzed the hydrogen peroxide synthesis using different PEDOT‐based electrodes. The study analyzed a commercially available PEDOT : PSS product, electropolymerized and chemically polymerized PEDOT formulations as thin‐film electrodes.^[20]^ Electrodes fabricated from the commercially available PEDOT : PSS formulation revealed the best performance with a current efficiency of 60–70 % and a production rate of 0.006 mg/min/cm^2^ at pH 2.

PEDOT : PSS is known to crystallize, increasing its conductivity and mechanical strength when exposed to sulfuric acid.[^21^, ^22^] This crystallization process allows the synthesis of stable and highly conductive PEDOT parts in different geometries.[^13^, ^17^, ^21^, ^23^] To the best of our knowledge, “acid‐treated” PEDOT has not been investigated as a catalyst for the oxygen reduction reaction. However, it poses the advantages as mentioned above over the native PEDOT : PSS formulations.

In the present publication, we analyze the catalytic performance of acid‐treated PEDOT : PSS in the oxygen reduction reaction to answer whether this type of PEDOT is a suitable candidate for the synthesis of hydrogen peroxide. Furthermore, we report the combination of a bottom‐up wet‐spinning process with the acid‐induced coagulation of PEDOT : PSS that enables the fabrication of porous tubular hollow fiber electrodes made from acid‐treated PEDOT : PSS in a continuous process.

Within the class of metal‐free catalysts, carbon nanotubes (CNTs) have been reported as an efficient catalyst for hydrogen peroxide synthesis. Due to their high catalytic activity, CNTs have been successfully applied as a catalytic coating for macroscopic carbon‐based electrodes.[^24^, ^25^] In 2014, Gendel *et al*. reported the fabrication of microtubular all CNT electrodes.^[26]^ The tubular geometry allows direct utilization as a gas diffusion electrode (GDE). Furthermore, it leads to a high surface‐to‐volume ratio and enables an increased packing density. Hydrogen peroxide was synthesized with a current efficiency of 48.9 % at −0.45 V (vs. SHE‐standard hydrogen electrode) and a production rate of 0.007–0.008 g/min/cm^2^. A significant challenge connected to tubular CNT electrodes poses limited mechanical stability, limited flexibility and contact resistances between the individual nanometer‐sized carbon building blocks.^[18]^

To overcome these drawbacks, we envision the development of composite electrodes made from highly conductive acid‐treated PEDOT : PSS with embedded CNTs. Thereby a synergetic improvement of the electrode performance should be achieved, combining the catalytic activity of CNTs with the flexible and highly conductive PEDOT : PSS matrix. In 2018, Chen *et al*.^[25]^ reported the modification of a carbon black GDE with a catalytic amount of PEDOT : PSS/CNT composites, which showed the first evidence for an improved hydrogen peroxide formation. To date, free‐standing PEDOT : PSS/CNT composites are not reported as electrodes for hydrogen peroxide production in literature. In the present publication, flat sheet and hollow fiber PEDOT : PSS/CNT composite electrodes with different CNT loadings have been fabricated, and the influence of the CNT addition on the material properties and their catalytic performance are evaluated.

## 2 Results and Discussion

### 2.1 Hollow Fiber Spinning

**Figure 1:**
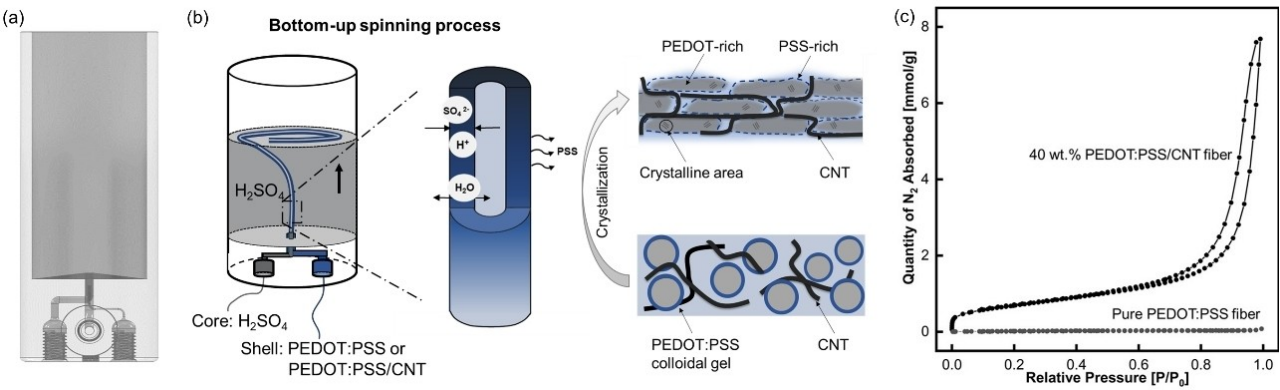
(a) CAD drawing of the 3D‐printed core‐shell spinning nozzle; (b) One‐step hollow fiber spinning process via sulfuric acid‐induced crystallization of a PEDOT : PSS suspension; (c) N_2_ isotherms at 77 K for PEDOT : PSS and PEDOT : PSS/CNT composite fibers.

The synthesis of PEDOT : PSS and PEDOT : PSS/CNT hollow fibers was performed using a bottom‐up wet‐spinning process. A unique 3D‐printed core‐shell spinning nozzle with a directly integrated coagulation/crystallization bath (see Figure 1(a)) was developed for fiber fabrication utilizing a PEDOT : PSS solution as the shell fluid and concentrated sulfuric acid as the coagulation/crystallization bath and the bore fluid (Figure 1(b)). The dimensions of the bore (1.0 mm in diameter) and the shell (3.0 mm in diameter) nozzle were chosen in a way, that on the one hand, blocking of the nozzle during the spinning process is avoided, and at the same time, the thickness of the PEDOT : PSS shell fluid is thin enough to ensure a fast and full solidification which is paramount for the formation of stable hollow fibers. With the given combination of nozzle dimension and flow rates of the shell and bore fluid, fibers can be fabricated continuously with a speed of 1.9 m/min. Upon exposure of the shell fluid to the sulfuric acid in the fiber lumen and the coagulation bath, the colloidally stable PEDOT : PSS suspension solidifies into a stable hollow fiber geometry. The solidification results from the crystallization of PEDOT by the partial dehydration of the suspension and the removal of negatively charged PSS, leading to less repulsion between the individual PEDOT domains (see Figure 1(b)).^[22]^ The crystallization of the utilized PEDOT : PSS suspension after acid treatment was confirmed using X‐Ray Diffraction Analysis (XRD) (see Figure S1). The XRD spectra of acid‐treated PEDOT : PSS revealed three pronounced reflection peaks at 2θ=6.1°, 11.7° and 26.2°. These peaks correspond to the (100) (200) and (010) planes of crystalline PEDOT : PSS and agree with literature reporting the crystallization of PEDOT : PSS in sulfuric acid.^[27]^ In contrast to this, the untreated PEDOT : PSS sample reveals no sharp and less pronounced reflection peaks, indicating the amorphous structure of this sample.[^22^, ^28^]

**Figure 2:**
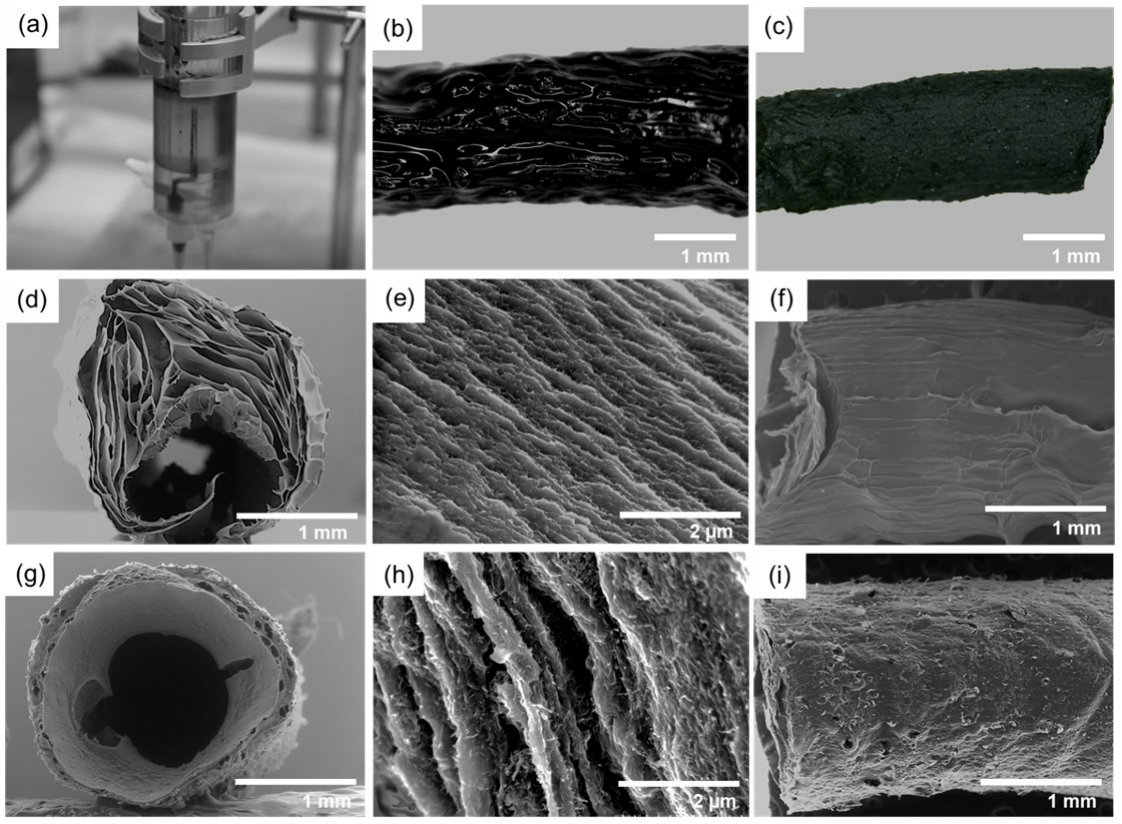
(a) Hollow fiber spinning process; Light‐microscopic image of (b) wet hollow fiber; (c) dry hollow fiber; Electron micrographs of the cross‐section and surface morphology of (d–f) PEDOT : PSS hollow fibers; (g–i) 40 wt % PEDOT : PSS/CNT composite hollow fibers.

Using the 3D‐printed spinning setup (see Figure 2(a)), we synthesized PEDOT : PSS and PEDOT : PSS/CNT composite fibers with 40 wt % CNT loading (see supporting videos SV1 and SV2). The CNTs were dispersed by ultrasound in the PEDOT : PSS suspension before spinning, ensuring an even distribution. Both fiber types are analyzed by N_2_ adsorption/desorption experiments to evaluate the proposed increased surface area and porosity due to the CNT addition. Figure 1(c) shows the isotherms for the PEDOT : PSS fiber and the PEDOT : PSS/CNT composite fiber. The isotherm of the composite fiber shows significant adsorption, whereas nearly no adsorption is detectable with the PEDOT : PSS fiber. The Brunauer‐Emmett‐Teller (BET) surface areas are determined to be 1.6 m^2^ g^−1^ for the PEDOT : PSS and 53.7 m^2^ g^−1^ for the composite fiber indicating an increase in surface area by a factor of 33.

Figure 2(b, c) shows the obtained fibers in the wet and the dry state. The pictures reveal the macroscopic fiber morphology, depicting flexible fibers with an even outer diameter (2.0 mm) in the wet state and a decreased diameter in the dry state (1.5 mm). When exposed to aqueous media, the polymer network of the fibers swells, generating more free volume within the polymer and thereby increasing the available area for electrochemical reactions.^[29]^

Electron microscopy was utilized to analyze the microscopic fiber morphology. The cross‐section images depict a macroporous composite hollow fiber in Figure 2(g) with a wall thickness of 200 μm and an outer fiber diameter of 2.0 mm. Compared to the symmetrical cross‐section of the composite hollow fiber, the PEDOT : PSS fiber has an unsymmetric structure with a varying wall thickness (Figure 2(d)). The high‐resolution cross‐section images of both fibers reveal a lamellar structure (Figure 2(e, h)). In the composite fiber (Figure 2(h)), a high quantity of evenly distributed CNTs forming an interconnected network is visible. The more uniform fiber shape and the absence of large defects may be attributed to the dispersed CNTs, creating a network during spinning, which stabilizes the shape of the shell fluid during the solidification process. Figure 2(f, i) shows the respective electron microscopy images of the fiber surface. In direct comparison, the surface of the composite hollow fiber in Figure 2(i) reveals an increased roughness and a specific porosity. The pure PEDOT : PSS fiber has, in contrast, a smooth surface without visible pores (see Figure 2(f)). In summary, we could report the successful synthesis of PEDOT‐based hollow fibers for the first time using a continuous bottom‐up sinning process.

### 2.2 Composite Characterization

To evaluate the effect of the CNT addition on the material properties, we fabricated acid‐treated flat sheets with CNT loadings between 0 wt % and 40 wt %. 40 wt % CNT loading poses the upper limit within this work as a higher concentration hinders the formation of stable fibers and flat sheets. The flat sheet geometry was chosen as it allows the fast and accurate analysis of different material properties. Concerning the desired application within an electrochemical cell, the conductivity and the capacitance were analyzed.

Figure S2 depicts the impact of different CNT loadings on the microscopic flat sheet morphology. The cross‐section of PEDOT : PSS is smooth and without any defects. By increasing the CNT loading from 5 wt % to 40 wt %, the PEDOT : PSS/CNT composites reveal a more pronounced layered structure with a densely interconnected fibrous CNT network. For all CNT loadings, a homogenous CNT distribution without any apparent aggregation within the PEDOT : PSS matrix is achieved. The surface of the composite flat sheets reveals an increased roughness compared to the PEDOT : PSS samples, which is in agreement with the observation made during hollow fiber synthesis. Raman spectral analysis was performed to investigate the immobilization of the CNTs into the PEDOT : PSS matrix and analyze the interactions between the two materials. Figure S3 shows the corresponding spectra of the individual materials and the composite. The spectrum of the composite shows three pronounced bands, with the band at 1424 cm^−1^ corresponding to the characteristic band of PEDOT. Compared to the pure PEDOT : PSS sample, the shoulder peaks disappear, which can be attributed according to literature to the high density of the CNT network.^[30]^ The bands at 1265 cm^−1^ and 1600 cm^−1^ correspond to the graphite E_2g_ (G) mode and the disordered A_1g_ (D) mode of CNTs, which shows the successful CNT immobilization in the PEDOT matrix. In comparison to the pure CNT sample, the peaks are shifted. According to literature, this indicates interactions between the materials which are likely based on π‐π interactions between the delocalized π bond network of CNTs and the thiophene rings of PEDOT backbone.^[31]^

**Figure 3:**
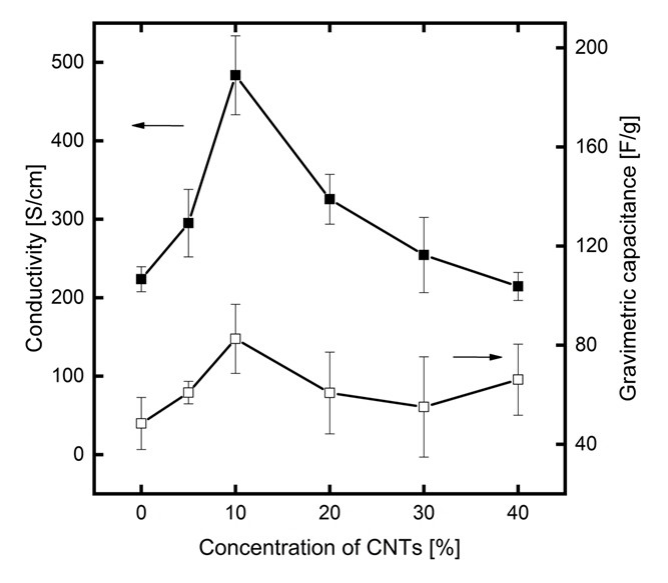
Conductivity and capacitance of PEDOT : PSS and PEDOT : PSS/CNT composite flat sheets with CNT loadings between 0 wt % and 40 wt % (Each point represents the mean value of at least three independent measurements. The error bars represent the respective standard deviation).

Figure 3 shows the impact on different CNT loadings between 0 wt % and 40 wt % on the conductivity and the capacitance. PEDOT rectangular flat sheets with a size of 4×4 mm were connected on each side using silver plates to minimize contact resistances (see Figure S4) to measure the conductivity. Acid‐treated pure PEDOT : PSS has a conductivity of 224 S/cm, indicating that the acid treatment indeed sharply improved the conductivity compared to its original PEDOT : PSS product, which has an initial conductivity of 0.3 S/cm.^[32]^ Within the interval between 0 wt % and 10 wt %, the conductivity increases by a factor of two to 484 S/cm. This increase can be attributed to the well‐dispersed CNTs forming conducting pathways within the composite material. The increasing loading of CNTs may help to build the network structure through the interpenetration of CNTs and PEDOT : PSS,^[23]^ forming the percolating path of CNTs in the polymer matrix,^[33]^ and the high tunnelling effect between CNT junctions. Due to the π‐π interaction of CNTs and thiophene ring, the charges become more delocalized on the PEDOT : PSS chains, leading to electronic density transfer from PEDOT to CNTs.^[34]^

A further increase in CNT loading leads to a decreasing conductivity. Between 10 wt % and 40 wt %, the conductivity continuously decreases towards the initial value of acid‐treated PEDOT : PSS. This decrease in conductivity may be attributed to the fact that, at such high concentrations, the contact resistances between the individual CNTs become dominant.^[35]^ The contact resistance between individual CNTs was also identified as the limiting factor in tubular all CNT electrodes, which show a conductivity of 20 S/cm.^[26]^ These contact resistances are reduced for CNT loadings within the composite material up to 10 wt % due to the highly conductive PEDOT : PSS matrix that surrounds and connects the individual CNTs.^[34]^ This optimum in conductivity for PEDOT : PSS/CNT composites with high CNT loadings has not been reported in the literature on composites with small CNT loadings.[^23^, ^35^]

In addition to the conductivity measurement, the influence of the CNT loading on the capacitance was analyzed. The measurement was performed in a specially designed test cell using a three‐electrode setup as depicted in Figure S5. The capacitance measurement reveals a similar trend observed for the conductivity, with the highest capacitance measured for the composite with a CNT loading of 10 wt %. Using this composite, the capacitance is increased by 70 % compared to the pure PEDOT : PSS sample. Furthermore, the capacitance is higher compared to a pure CNT sample (22.38±7.87 F/g). A possible explanation for this increase poses the combination of the double‐layer capacitance originally from CNTs and the pseudo‐capacitance from PEDOT : PSS. Furthermore, the open mesoporous CNT network improves the accessibility of the electrode/electrolyte interface and enables quick charge transport into the entire composite electrode, thereby allowing fast and efficient charge/discharge cycles.[^36^, ^37^] At higher CNT loadings up to 30 wt % the capacitance decreases slightly. This decrease in capacitance may be attributed to the overall decreasing conductivity or CNT agglomeration. For the highest CNT loading, the measurements indicate a second increase in capacitance. However, with the current measurement method, the effect is in the same order of magnitude as the measurement error. To date, the reason for this increase is not apparent and the detailed investigation of this phenomenon poses an ongoing challenge, which we would like to address in future work. In summary, the results highlight the synergistic improvement of the electrical properties within the composite material, leading to an improved conductivity and capacitance compared to the individual starting materials.

A significant drawback of nanocarbon materials as CNTs poses their limited processability into stable free‐standing electrodes. In contrast to the high mechanical strength of the individual CNTs macroscopic, all CNT electrodes are self‐supporting networks of entangled CNT assemblies arranged randomly and held together in a buckypaper or a tubular structure by van der Waals interactions between the individual CNTs.[^38^, ^39^, ^40^] Regarding electrochemical reactors beyond the lab‐scale, the mechanical stability of those nanocarbon electrodes is a challenge. Within the present work, individual CNTs are embedded into a conductive PEDOT : PSS matrix to increase durability and flexibility compared to pure CNT material. Table 1 presents the mechanical properties of the developed free‐standing PEDOT : PSS and PEDOT : PSS/CNT composite electrodes directly compared to a free‐standing all CNT electrode.^[41]^ The addition of CNTs to the PEDOT : PSS matrix leads to a reduction in Young's Modulus, break stress and break strain. In direct comparison to the mechanical properties of all CNT electrodes, the PEDOT : PSS/CNT composite reveals significantly improved mechanical properties. Due to the addition of PEDOT : PSS as a conductive matrix, the Young's Modulus and the break strain of the composite are increased to a factor of 3.6 and 1.9, thereby achieving a superior combination of flexibility and toughness. These results highlight the suitability of PEDOT : PSS as a polymeric binder stabilizing the otherwise loosely interconnected CNT network.

**Table 1:** Mechanical properties of free‐standing electrodes (the mean value and standard deviation were calculated based on five independent measurements).

Sample	Young's Modulus, GPa	Break Stress, MPa	Break Strain, [%]
PEDOT : PSS	0.39±0.21	34.21±11.78	6.38±2.18
40 wt % composite	0.29±0.16	15.72±1.94	4.86±1.34
CNT	0.08±0.006	1.33±0.40	2.60±0.26

### 2.3 Oxygen Reduction Reaction

**Figure 4:**
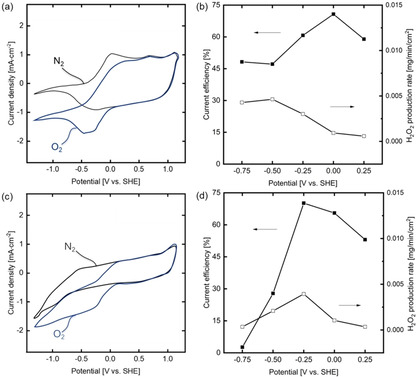
Cyclic voltammetry of flat sheet electrodes under N_2_ and O_2_ gassing: (a) PEDOT : PSS; (c) 40 wt % PEDOT : PSS/CNT composite. Current efficiency and H_2_O_2_ production rate were calculated from potentiostat experiments at five different potentials: (b) PEDOT : PSS; (d) 40 wt % PEDOT : PSS/CNT composite.

The catalytic activity of acid‐treated PEDOT : PSS and PEDOT : PSS/CNT composite electrodes towards the oxygen reduction reaction was analyzed in an H‐cell setup by cyclic voltammetry (CV) and chronoamperometry (CA) experiments. Figure 4 depicts the respective cyclic voltammograms (CVs) under oxygen and nitrogen gassing between −1.4 V and 1.3 V (vs. SHE) at a scan rate of 10 mV/s for a PEDOT : PSS (see Figure 4(a)) and a PEDOT : PSS/CNT electrode with a CNT loading of 40 wt % (Figure 4(c)). To determine the suitable potential range for the electrochemical reduction of oxygen and mitigate hydrogen evolution, CVs were also measured under nitrogen gassing. In the forward scan direction from positive towards negative potentials ( −1.3 V to 1.2 V (vs. SHE)), no significant current flow can be detected under nitrogen gassing for the PEDOT : PSS electrode. Only a small current peak can be observed between 0.1 and −0.5 V (vs. SHE). At lower potentials, the current density decreases. This behavior can be explained by the transition of the PEDOT : PSS electrode from the conductive oxidized into the nonconductive reduced form.^[42]^ The results indicate that pure PEDOT : PSS electrodes do not significantly favor the hydrogen evolution reaction, which is in agreement with literature on acid‐treated vapor‐phase polymerized PEDOT electrodes.^[43]^ In contrast to the PEDOT : PSS electrode, the PEDOT : PSS/CNT composite electrode shows an increasing current density for potentials between −0.5 and −1.4 V (vs. SHE), which indicates the activity of the embedded CNTs for the hydrogen evolution reaction within this potential range. To avoid the loss of the electrode conductivity and suppress the hydrogen evolution reaction, the potential for the oxygen reduction reaction should be located above −0.5 V (vs. SHE).

In contrast to the cyclic voltammetry under nitrogen gassing, the experiment in the presence of oxygen shows significantly higher current densities within the low potential range for both electrode types. This increased current density indicates the catalytic activity of acid‐treated PEDOT : PSS with an onset potential of 0.1 V (vs. SHE). Within the potential range between 0.1 and −0.5 V (vs. SHE), both electrodes show an increasing current density to a final value of −1.6 mA/cm^2^ for the PEDOT : PSS and −1.3 mA/cm^2^ for the composite electrode. For more negative potentials, the current decreases for the PEDOT : PSS electrode can be attributed to the loss in conductivity and activity. In contrast to this, the current density increases steadily for the composite electrode, which can be explained by the onset of the hydrogen evolution reaction measured under nitrogen gassing for potentials lower than −0.5 V (vs. SHE). The cyclic voltammetry experiments reveal the activity of both electrode types towards the oxygen reduction reaction. However, the selectivity towards the desired two‐electron transfer forming hydrogen peroxide and its production rate cannot be extracted from these experiments.

To evaluate the selectivity of the developed electrodes towards the two‐electron ORR to hydrogen peroxide, chronoamperometry experiments were performed at different potentials (0.25 and −0.75 V vs. SHE) for 180 min. Figure S6 shows the development of the hydrogen peroxide concentration in the electrolyte. For all tested potentials and both electrode types, the hydrogen peroxide concentration increases linearly with time, proving that the electrodes can produce hydrogen peroxide at a constant rate. The current efficiency and the hydrogen peroxide production rate were calculated based on these experiments to analyze the electrode performance within the given potential range and benchmark it against other PEDOT : PSS and CNT‐based electrodes from literature (see Figure 4(b, d)).

The PEDOT : PSS electrode shows the highest current efficiency of 71 % at a potential of 0 V (vs. SHE), which decreases continuously towards 46 % at a potential of −0.5 V (vs. SHE). After this decrease, the current efficiency stays constant for the lowest potential. This decrease may be attributed to the increasing extent of side reactions or limited oxygen solubility in the electrolyte. In contrast to the decreasing efficiency, the hydrogen peroxide production rate continuously increases up to 0.005 mg/min/cm^2^ at a potential of −0.50 V (vs. SHE) and stays nearly constant until −0.75 V (vs. SHE). The increase can be explained by the more potent driving force due to the increasing potential. However, the reduction of PEDOT : PSS at −0.75 V (vs. SHE) combined with increasing side reactions limited this increase. For the first time, the results show that acid‐treated PEDOT : PSS is a suitable catalyst for the electrochemical synthesis of hydrogen peroxide.

Figure 4(d) shows the development of the current efficiency and production rate for a PEDOT : PSS/CNT composite electrode with a CNT loading of 40 wt %. The composite electrode shows an optimum current efficiency of 71 % and a production rate of 0.004 mg/min/cm^2^ at a potential of −0.25 V (vs. SHE). For lower potentials, the current efficiency strongly decreases towards 0 %. This substantial decrease can be explained by the pronounced activity towards the hydrogen evolution (see Figure 4(c)) within this potential range in combination with the reduction of PEDOT : PSS. The composite electrode reveals a higher current efficiency of 71 % at a potential of −0.25 V (vs. SHE) in comparison to the pure PEDOT : PSS electrode (60.9 %). Thereby the hydrogen peroxide production rate at the point of the highest efficiency is increased by a factor of 1.3 to 0.004 mg/min/cm^2^.

This superior combination of high selectivity and activity highlights the synergistic effects of incorporating catalytic active CNTs into the flexible, conductive, and catalytic active PEDOT : PSS matrix. This leads to an improved electrode performance compared to pure PEDOT : PSS electrodes and comparable catalytic activity to tubular pure CNT gas diffusion electrodes.[^20^, ^26^]

Using this highly efficient and active composite electrode material, the suitability of wet spun composite hollow fibers with a CNT loading of 40 wt % (see Figure 2(g–i)) is analyzed by chronoamperometry at the optimum potential of −0.25 V (vs. SHE).

The hollow fiber electrode reaches a current efficiency of 54.3 % and a significantly increased hydrogen peroxide production rate of 0.011 mg/min/cm^2^. The flat sheet electrode experiments highlighted the improvement in the hydrogen peroxide production rate using the composite electrode compared to the pure PEDOT electrode. This increase can be attributed to the synergetic effect of the immobilization of highly catalytic active CNTs into the conductive and also catalytic active PEDOT : PSS matrix, which on the one hand increases the number of active sites and the electrode conductivity and, on the other hand, leads to an overall higher intrinsic surface area. The pronounced performance increase by the transition from the flat sheet composite electrode towards a hollow fiber electrode can be attributed to the fiber morphology. The hollow fiber consists of a macroporous lamellar wall with a higher thickness compared to the flat sheet. Due to the open porous structure, the entire material is accessible for the electrochemical reaction, thereby enabling high production rates.

**Figure 5:**
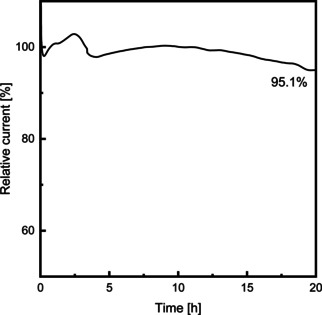
Long‐term durability test in ORR at −0.25 V vs. SHE for a PEDOT : PSS/CNT composite fiber with a CNT loading of 40 wt %.

Besides the high activity of the composite hollow fiber, the long‐term stability of an electrode poses a vital parameter with respect to the implementation in an electrochemical process. Figure 5 depicts the development of the current during a long‐term experiment at a constant potential of −0.25 V (vs. SHE). The composite fibers exhibited a stable current with a high relative current retention of 95.1 % after a 20 h experiment, indicating sufficiently long‐term stability of the wet‐spun fiber electrode.[^9^, ^44^]

## 3 Conclusion

A facile one‐step fabrication platform for the continuous production of PEDOT : PSS and PEDOT : PSS/CNT composite hollow fibers with a CNT loading of up to 40 wt % was developed. The process is based on a 3D‐printed core‐shell spinneret for bottom‐up spinning, which allows for stable fiber formation by the acid‐induced coagulation and crystallization of the PEDOT : PSS shell fluid. The addition of CNTs into the PEDOT : PSS matrix has been shown to improve the spinning process allowing the fabrication of evenly shaped porous hollow fibers. Mechanical analysis of the PEDOT : PSS/CNT composites reveal a significantly increased Youngs Modus (factor 3.8) and Tensile Strength (factor 1.8) compared to conventional tubular all CNT electrodes. Furthermore, the addition of CNT leads to an increased inner surface area. The addition of CNTs increases the conductivity and capacitance of the composite material with an optimum CNT loading of 10 wt %. Electrochemical experiments prove the suitability of acid‐treated PEDOT : PSS for the electrochemical synthesis of hydrogen peroxide. Under optimized conditions, the fabricated PEDOT : PSS/CNT hollow fiber electrodes produce hydrogen peroxide with a production rate of up to 0.01 mg/min/cm^2^ with a faradaic efficiency of 54 %. Furthermore, the electrode has proven to be long‐term stable with a current retention of 95 % during 20 h of hydrogen peroxide synthesis. This type of electrode has excellent potential for application in larger electrochemical modules due to the combination of high mechanical strength and high catalytic activity and stability. The fabrication process offers a large degree of flexibility and can be adapted to different fiber sizes and carbon‐based or metal‐based additives, enabling new PEDOT : PSS composite hollow fibers in the future. Due to the tubular and porous nature of the hollow fibers, a potential application could be within the field of gas diffusion electrodes or electroactive membranes combining electrochemical reactions and a separation task within one process step.

## Experimental Section

### Materials

Clevious PH1000 PEDOT : PSS solution (Heraeus, 1.3 wt % in water), multiwalled carbon nanotubes (Bayer Material Science, purity>99 wt %, diameter: 4–13 nm, length: >1 μm), Triton X‐100 (Sigma Aldrich, pure), phosphate‐buffered saline (PBS) (Sigma Aldrich), sulfuric acid (Sigma Aldrich, 97 %), sodium sulfate (Sigma Aldrich, purity>99.0 %), hydrogen peroxide (Sigma Aldrich, 30 %) and ammonium metavanadate (Sigma Aldrich, purity>99.0 %) were used without any further purification.

### Preparation of the PEDOT : PSS/CNT Solution

An aqueous dispersion of CNT with a concentration of 15 mg/g was prepared by the addition of the corresponding amount of CNT to deionized water containing 1 wt % Triton X‐100. The mixture was vigorously stirred for 30 min, followed by 3 h of sonication (Hielscher, UP200S, cycle 0.8, amplitude 65 %). In the following experiments, different amounts of this stock solution were added into PEDOT : PSS solution to achieve final CNT concentrations of 5 wt %, 10 wt %, 20 wt %, 30 wt %, and 40 wt %.

Hollow Fiber Spinning: The hollow fiber fabrication was performed via a bottom‐up spinning process in a self‐design 3D‐printed core‐shell spinneret (see Figure 1(b)). The core‐shell consists of two concentric channels with a diameter of 1.0 mm (core fluid) and 3.0 mm (shell fluid). The shell fluid (PEDOT : PSS or PEDOT : PSS/CNT solution) was fed into the spinneret with a flow rate of 12 ml/min, while sulfuric acid (97 %) was supplied as the core fluid with a flow rate of 5 mL/min. During fiber spinning, the fibers were spun into a coagulation bath containing sulfuric acid with the same concentration as the core fluid. After a residence time of one minute in the coagulation bath, the fibers were washed extensively with water to remove excess sulfuric acid.

Flat Sheet Fabrication: Flath sheets with an identical composition of the hollow fibers were prepared via drop‐casting 0.5 g PEDOT : PSS or PEDOT : PSS/CNT solution onto a glass slide followed by evaporation of the remaining water at 50 °C for 3 h. Subsequently, the flat sheets were immersed in 97 wt % sulfuric acid for 1 min and extensively washed with deionized water.

Morphology and Physical Characterization: The morphologies of the PEDOT : PSS and PEDOT : PSS/CNT samples were analyzed by field emission scanning electron microscopy (FeSEM Hitachi S4800) and scanning electron microscopy (SEM) a TM4000Plus Hitachi Tabletop Microscope. X‐Ray Diffraction (XRD) analysis was performed using an Empyrean X‐ray Diffractometer from PANalytical equipped with a Cu‐anode. The analyses were performed with the following parameters: U=40 kV, I=40 mA, λKα1=0.1540 nm, 4°–45° at room temperature. The specific surface area of PEDOT : PSS and PEDOT : PSS/CNT hollow fiber was determined by N_2_ adsorption/desorption at 70 K. The measurements were carried out using a Quantachrome Quadrasorb SI instrument. The samples were degassed under vacuum at a temperature of 250 °C and maintained for 12 h before performing the measurement. The specific surface area was calculated using the Brunauer‐Emmett‐Teller (BET) theory.

### Conductivity and Capacitance Measurements

The conductivity of PEDOT : PSS and PEDOT : PSS/CNT flat sheets was measured by the two‐point method, ensuring a reproducible good electrical connection by two silver plates pressed onto each end of the thin film. Prior to each experiment, the thickness of each individual film and the distance between the silver plates was measured. A schematic drawing of the conductivity measuring cell is depicted in Figure S4. The electrochemical cell for the capacitance measurement is shown in Figure S5. The measuring cell consists of two Fluorine doped Tin Oxide (FTO) glasses (50 mm×50 mm×2.2 mm) acting as the working electrode (WE) and counter electrode (CE) pressed to the top and the bottom of a PDMS slab (0.5 mm thickness) with a cylindrical opening (0.75 mm diameter) forming the measuring cell. The bottom FTO glass was equipped with a PEDOT : PSS or PEDOT : PSS/CNT flat sheet. Prior to the experiment, the cell was filled with PBS buffer solution (1X, pH 7.4), and a standard hydrogen reference electrode (SHE) (Mini HydroFlex® from gaskatel) was inserted from the side into the measuring cell. The capacitance of the thin films was measured by cyclic voltammetry (CV) in a potential range between 0 V–0.5 V (vs. SHE), and a scan rate is 50 mV/s for 5 cycles. The obtained cyclic voltammetry curve (last cycle) was used to calculate the gravimetric capacitance C_M_ (F/g) based on the following equation:

$$C_{M}=\frac{1}{\Delta V\;\cdot \;k\;\cdot \;m}\;\cdot \;\int_{\nu }^{\nu +\delta v}idV$$

V is the lower starting voltage (V vs. RHE), ΔV is the potential window (V vs. RHE), k is the scan rate (mV/s), m is the electrode mass (g), and i is the current (A).

### Tensile Experiments

Tensile experiments were performed on a Zwick Roell servohydraulic testing machine equipped with an Xforce P load cell with a maximum capacity of 100 N. The vertical tensile test measurement was carried out at 21 °C and 55 % relative humidity. Specimens were tested at a strain rate of 30 mm/min. The data present an average of 5 specimens.

### Raman Spectroscopy

Raman measurements were carried out using a Brucker RFS 100/S system. The 1064 nm line of a Nd: YAG laser was used as the excitation source in the region of 50–3600 cm^−1^.

### Electrode Preparation

Flat sheet electrodes were build using rectangular PEDOT : PSS and PEDOT : PSS/CNT composite flat sheets (1×1 cm), which were fabricated as described above. The flat sheet was glued with a carbon‐based conductive glue (Leit‐C, Sigma Aldrich) to a titanium plate (1.5×2 cm). After drying, the uncovered titanium plate and the excess of the PEDOT : PSS flat sheet was sealed with hot glue, forming a round working electrode with a surface area of 1.13 cm^2^ and avoiding direct contact between the electrolyte and the titanium plate (see Figure S7). During the experiment, the electrodes were contacted at the top of the titanium plate. Tubular electrodes were fabricated by inserting a thin titanium wire (0.2 mm diameter) coated with conductive glue (Leit‐C, Sigma Aldrich) into a PEDOT : PSS/CNT hollow fiber. After drying, the bottom opening of the hollow fiber was sealed with a drop of hot glue. The top part of the electrode was covered with shrinking tubing. Thereby the free fiber length was set to 3–4 mm, and contact between the uncoated titanium wire and the electrolyte is avoided (see Figure S7). During the electrochemical experiment, 4 electrodes were bundled and connected at the top of the titanium wire to the power source. The exact surface area of each electrode was investigated after the experiment via electron microscopy.

### Electrochemical Experiments

The analysis of the oxygen reduction reaction (ORR) was performed using a three‐electrode setup in an H‐cell filled with 80 mL of 50 mM sodium sulfate solution at pH 3 (Figure S7). A cation exchange membrane was implemented to separate the cathodic and anodic compartments. A mercury‐sulfate electrode (Hg/Hg_2_SO_4_) in 0.5 mol/L Hg_2_SO_4_ placed near to the PEDOT : PSS working electrode (surface area of each flat sheet electrode: 1.13 cm^2^, surface area for tubular electrodes: 0.55 cm^2^) was used as a reference electrode (RE). A titanium mesh coated with platinum was used as a counter electrode in the anodic compartment. The ORR was performed for different potentials under nitrogen or oxygen gassing. Pure nitrogen or oxygen was supplied to the solution and the electrodes via a gas inlet of the H‐cell equipped with a porous gas diffusor. To ensure the saturation of the electrolyte with oxygen or nitrogen, the measuring cell was bubbled with the respective gas for 20 min before the experiment. H_2_O_2_ was quantified photometrically at 450 nm based on the formation of peroxovanadium cation by the ammonium metavanadate reaction with H_2_O_2_ in an acidic solution.^[45]^ The ammonium metavanadate solution was prepared by dropwise addition of 10 mL sulfuric acid (9 mol/L) to 1.7547 g ammonium metavanadate. The solution was stirred for one hour at 50 °C. After complete dissolution, the solution was cooled down to room temperature and diluted with DI water was to a final volume of 250 mL. Samples of 2 mL were taken in regular time intervals from the cathodic compartment of the H‐cell and mixed immediately with 0.8 mL ammonium metavanadate solution. The samples were stored in the fridge until the UV/VIS‐Spectrometer measurement. The calibration curve (see Figure S8) was measured with standard solutions prepared by diluting 30 % H_2_O_2_ solution to 2.5 mg/L, 5 mg/L, 10 mg/L, 25 mg/L, 50 mg/L, 100 mg/L and 200 mg/L. The corresponding H_2_O_2_ concentration was calculated using the determined calibration curve. The measured amount of synthesized H_2_O_2_ in combination with the measured current flow during the ORR was utilized to calculate the current efficiency (CE) of the process according to the following equation:^[25]^

$$CE=\frac{n\cdot F\;\cdot {\;C}_{H_{2}O_{2}}\;\cdot \;V}{M_{H_{2}O_{2}}\;\cdot \;I\;\cdot \;t}\times 100\;\%$$

where n is the electron transfer number in ORR (n=2), F is the Faraday constant (96485 C/mol), C _H2O2_ and M _H2O2_ is H_2_O_2_ concentration in the electrolyte and the molecular mass of H_2_O_2_ (34 g/mol).

## Conflict of interest

The authors declare no conflict of interest.

## Supporting information

As a service to our authors and readers, this journal provides supporting information supplied by the authors. Such materials are peer reviewed and may be re‐organized for online delivery, but are not copy‐edited or typeset. Technical support issues arising from supporting information (other than missing files) should be addressed to the authors.

Supplementary
