# Supplementary material for: Wet‐Spun PEDOT/CNT Composite Hollow Fibers as Flexible Electrodes for H2O2 Production
Source: ChemElectroChem. 2021 May 4;8(9):1665–73. doi: 10.1002/celc.202100237 (PMC8251878; doi:10.1002/celc.202100237)
Supplement: Supplementary file 1 — Supplementary [file CELC-8-1665-s001.pdf]

ChemElectroChem

Supporting Information

Wet-Spun PEDOT/CNT Composite Hollow Fibers as Flexible Electrodes for H_2O_2 Production**

Qing Cui[+], Daniel Josef Bell[+], Siqi Wang, Mojtaba Mohseni, Daniel Felder, Jonas Lölsberg, and Matthias Wessling*

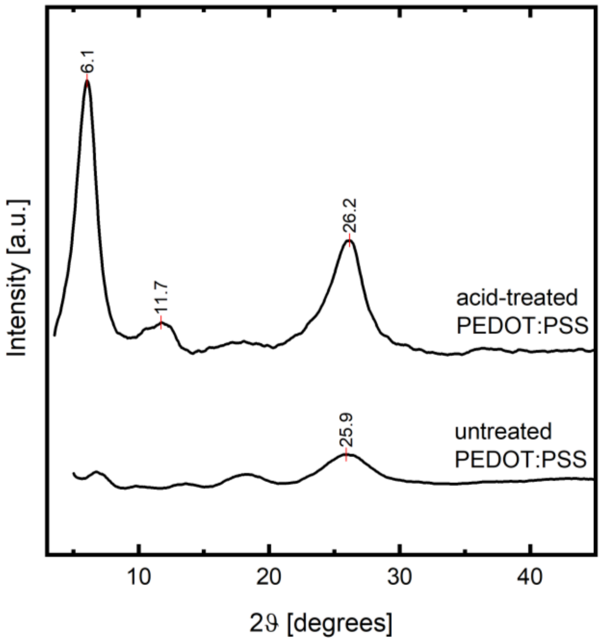

Figure S1. XRD curves of PEDOT:PSS before and after the acid treatment.

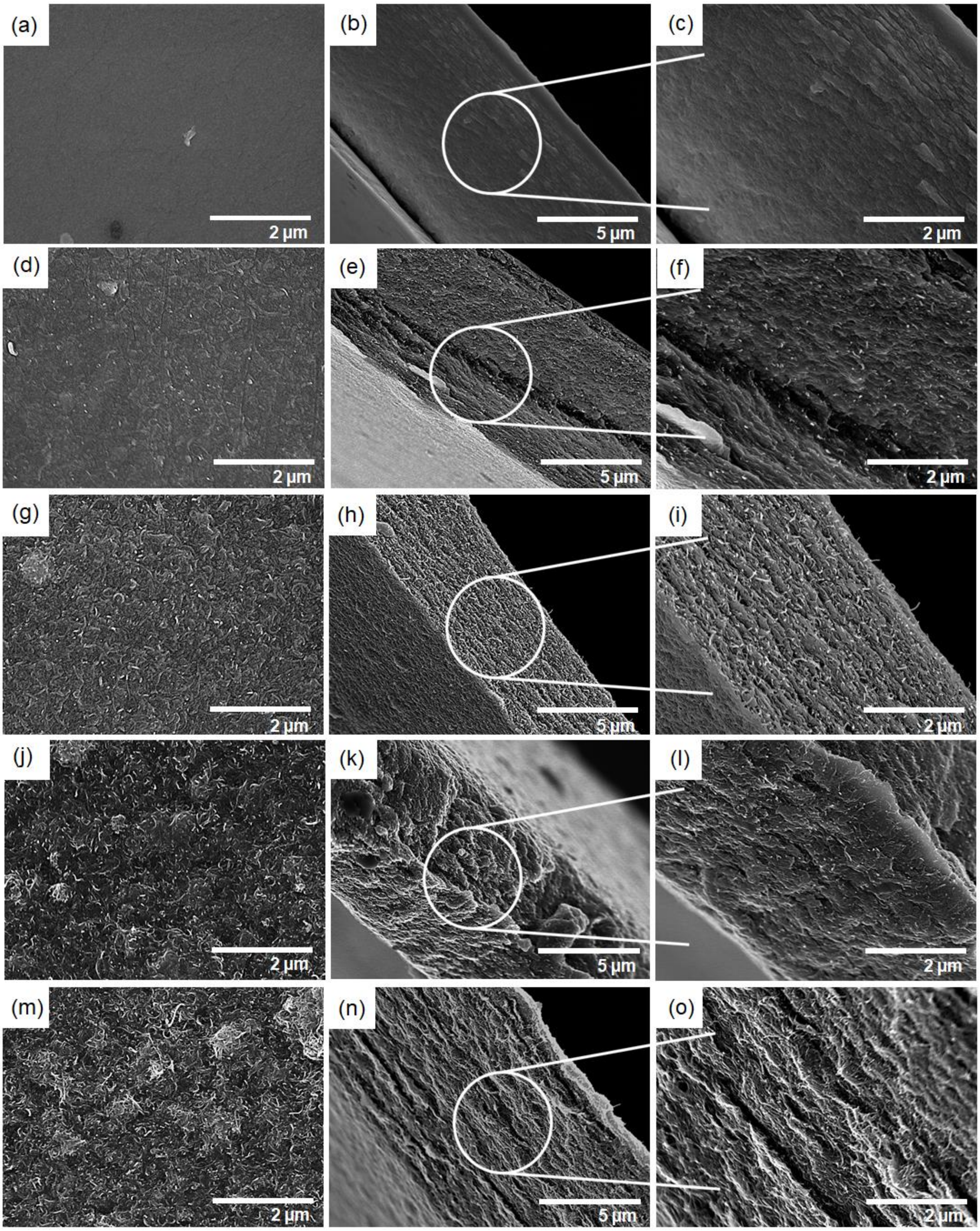

Figure S2. Electron micrographs of PEDOT:PSS (a) and PEDOT:PSS/CNT composites (d-o). (a) surface of PEDOT:PSS; (b, c) cross-section of PEDOT:PSS; (d) surface of 5% PEDOT: PSS/CNT composite; (e, f) cross-section of 5% PEDOT:PSS/ CNT composite; (g) surface of 10% PEDOT:PSS/CNT composite; (h, i) cross-section of 10% PEDOT:PSS/ CNT composite; (j) surface of 30% PEDOT:PSS/CNT composite; (k, l) cross-section of 30% PEDOT:PSS/CNT composite; (m) surface of 40% PEDOT:PSS/CNT composite; (n, o) cross-section of 40% PEDOT:PSS/CNT composite.

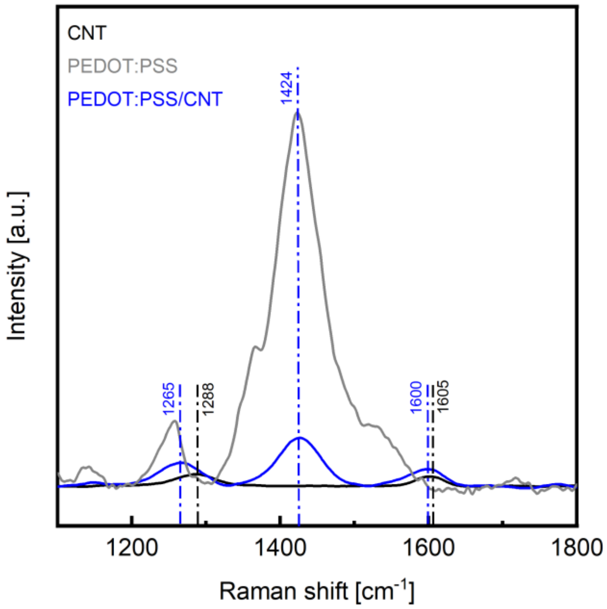

Figure S3. Raman spectra of PEDOT:PSS (grey), CNT (black) and PEDOT:PSS/CNT composite material with 40 wt.% CNT (blue).

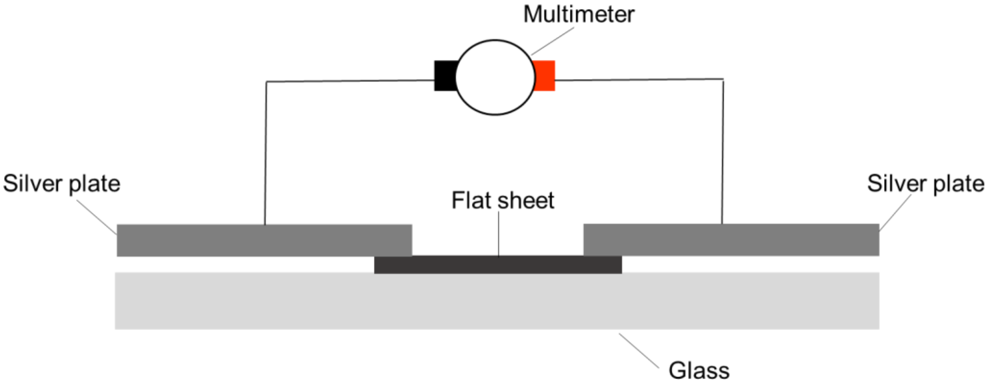

Figure S4. Conductivity measurement set-up.

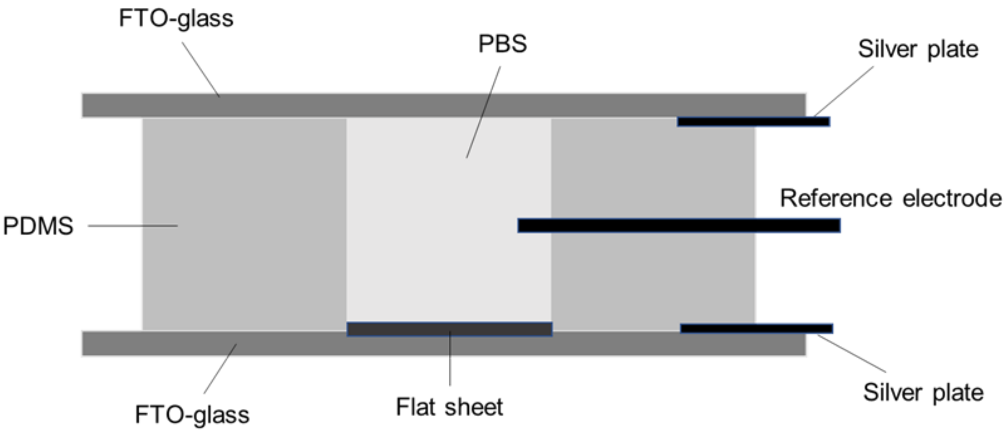

Figure S5. Capacitance measurement set-up.

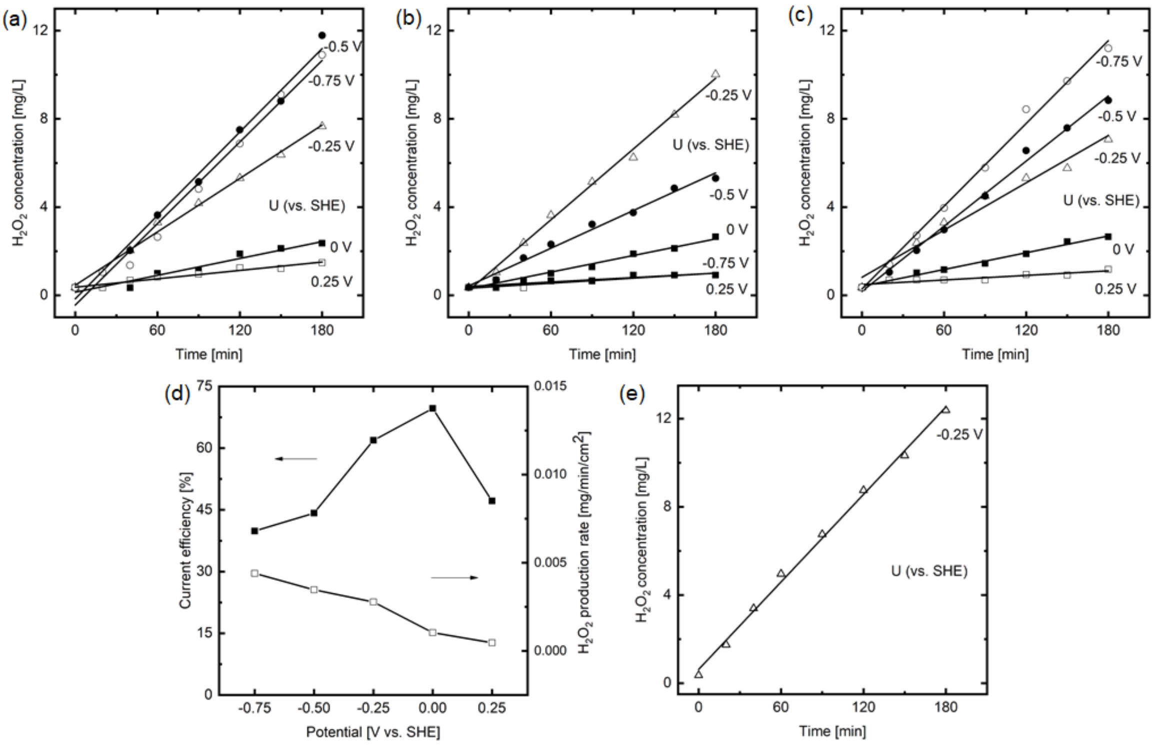

Figure S6. Hydrogen peroxide concentration increase at different potentials for PEDOT:PSS and PEDOT:PSS/CNT electrodes: (a) PEDOT: PSS flat sheets; (b) 40% PEDOT: PSS/CNT composite flat sheets; (c) 10% PEDOT:PSS/CNT composite flat sheets; (e) 40% PEDOT: PSS/CNT composite hollow fibers; (d) Current efficiency and hydrogen peroxide production rate at five potentials of 10% PEDOT:PSS/CNT composite flat sheets.

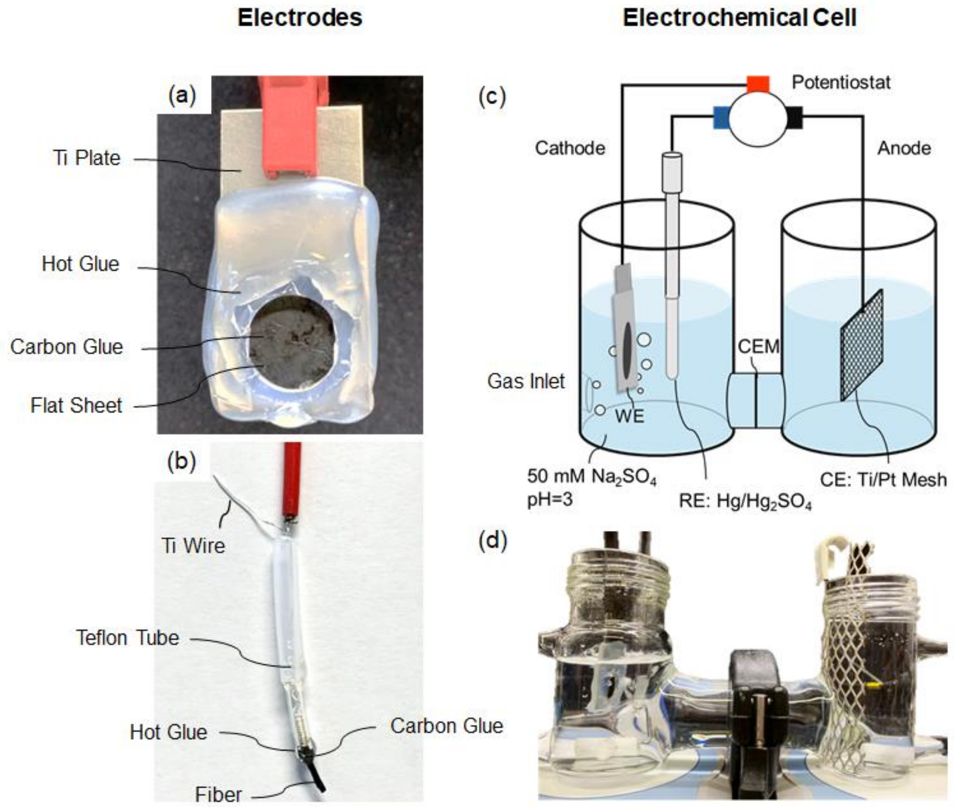

Figure S7. Photo of PEDOT:PSS/CNT composite working electrodes (a) flat sheet, (b) hollow fiber. Three-electrode set-up in a divided H-cell for the electrochemical experiment, cell divided by cation exchange membrane; CE: counter electrode, RE: reference electrode, WE: working electrode (c) schematic illustration, (d) photo of the set-up.

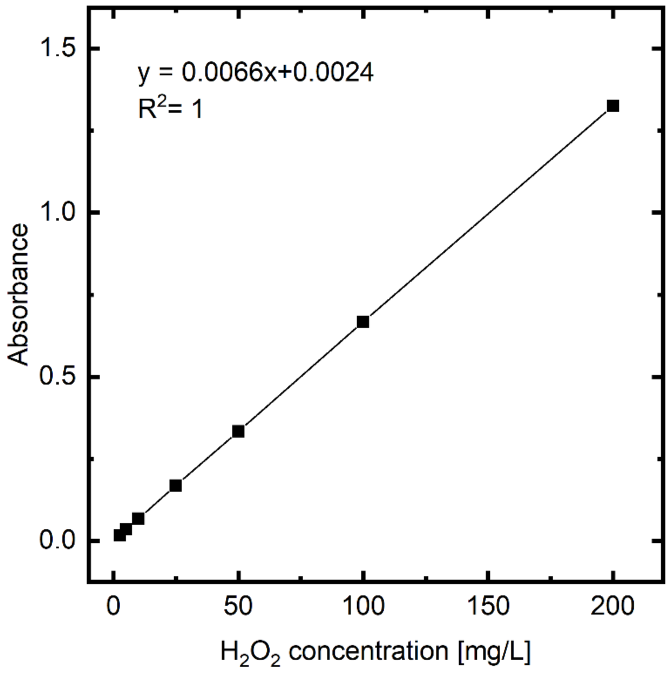

Figure S8. Calibration curve to quantify H_2O_2 photometric at 450 nm.
